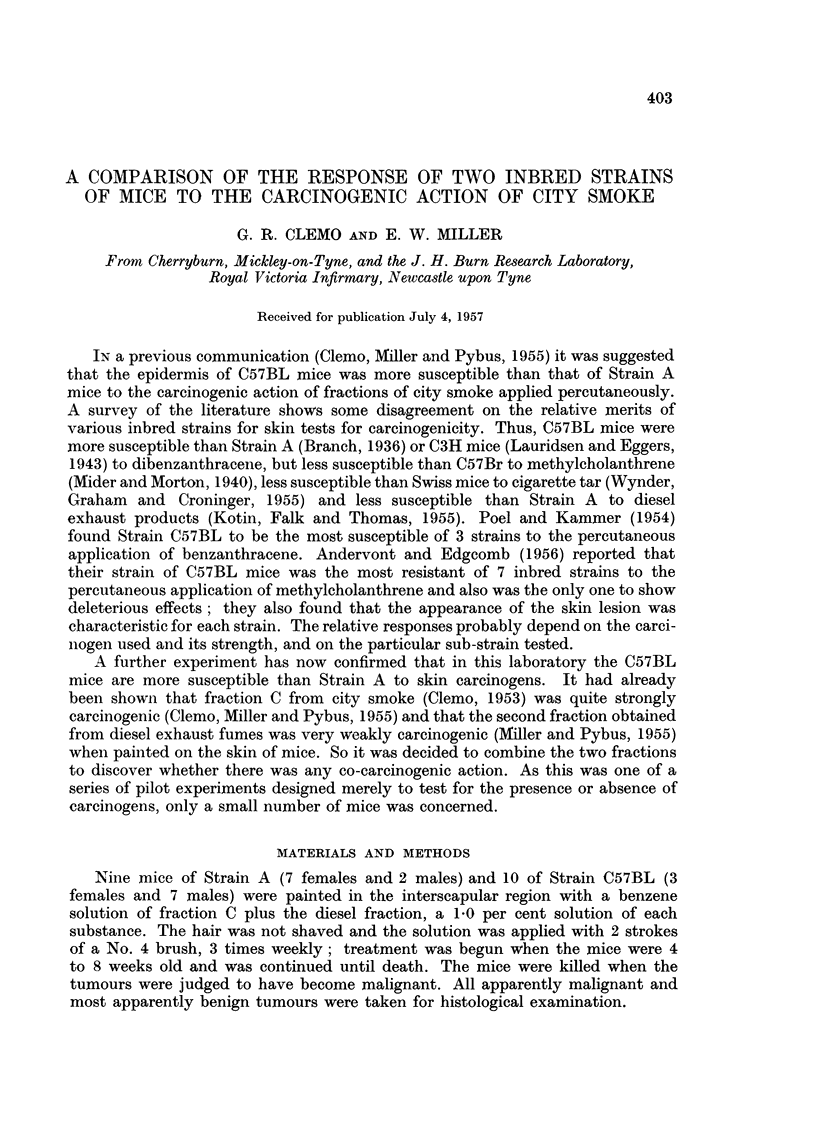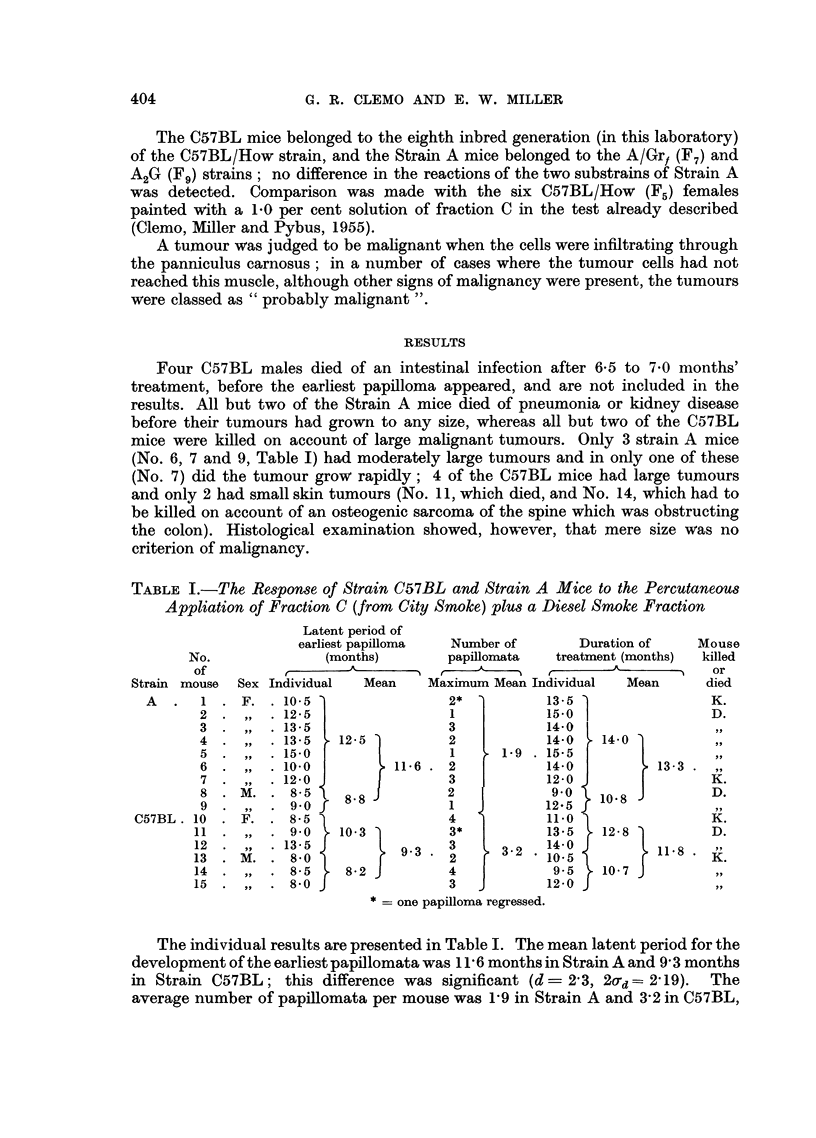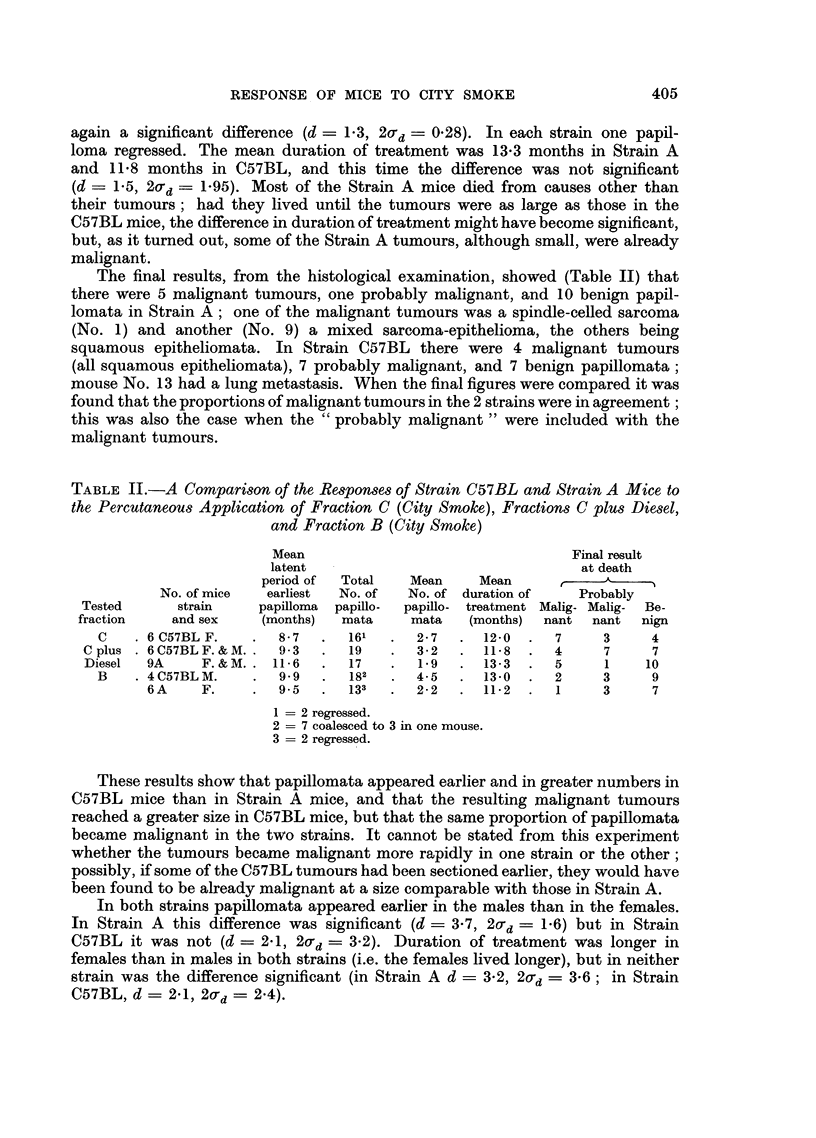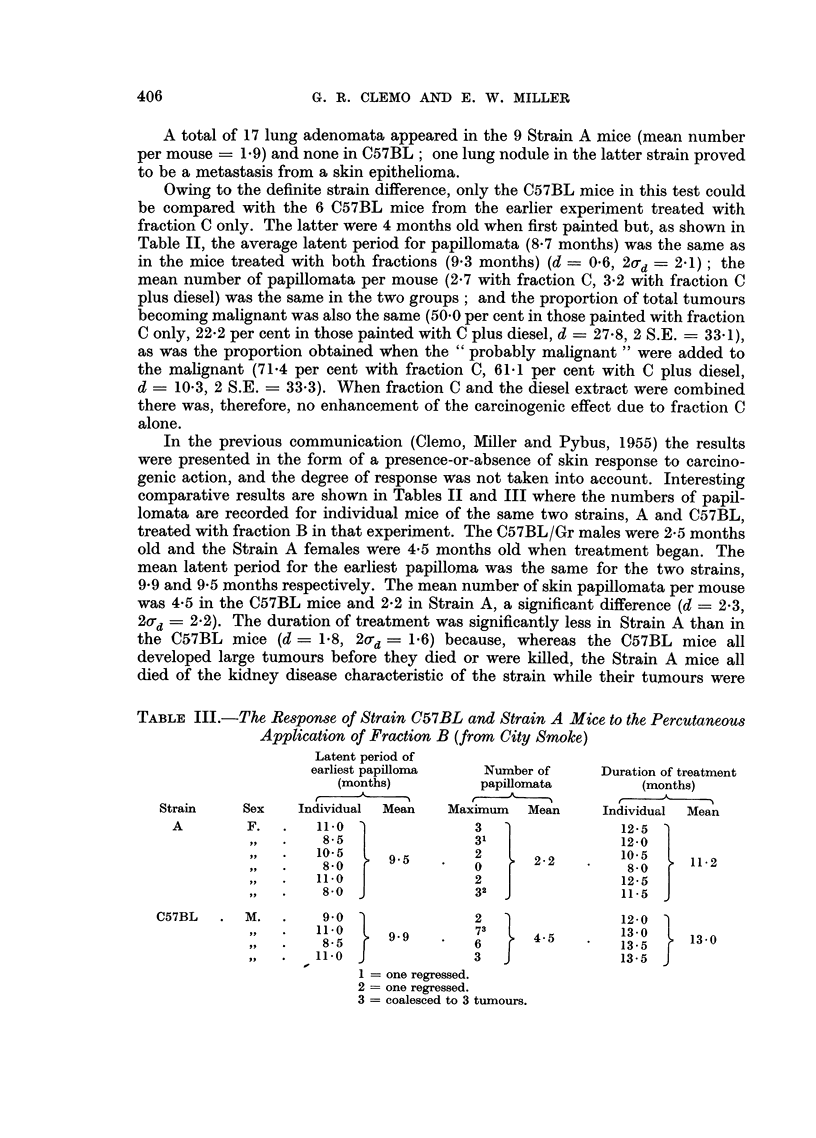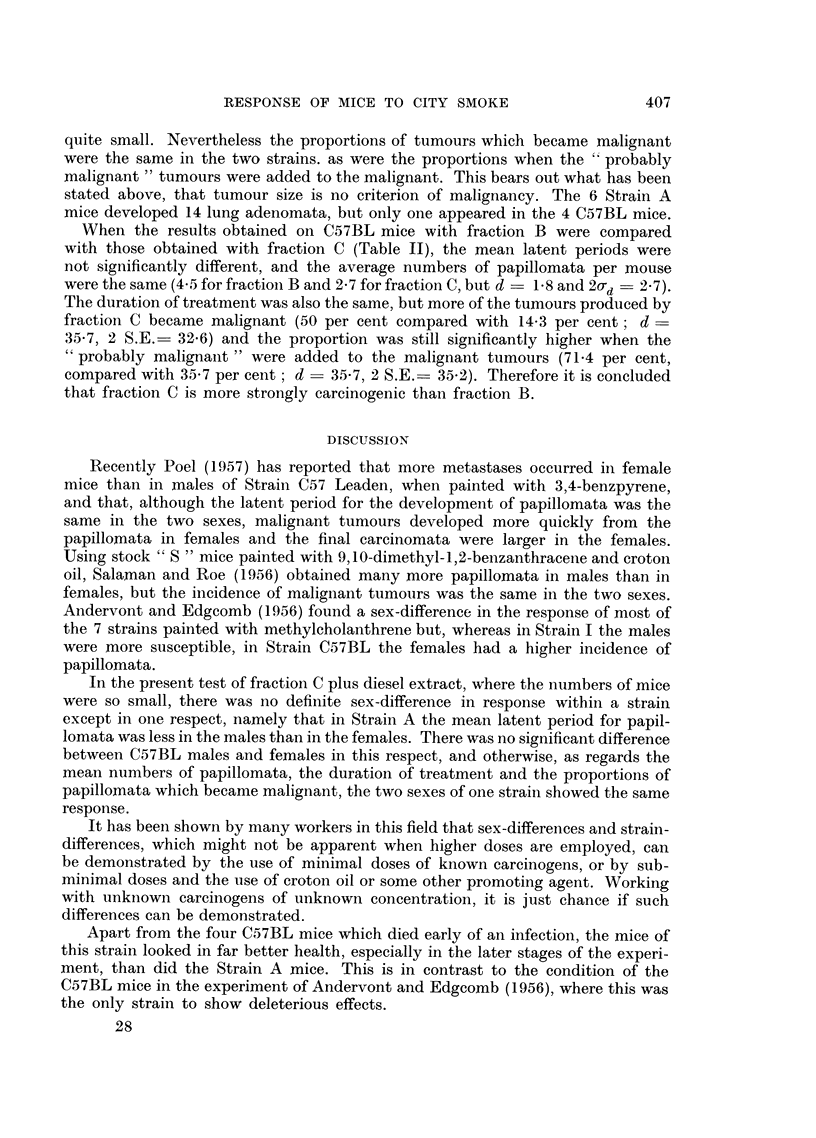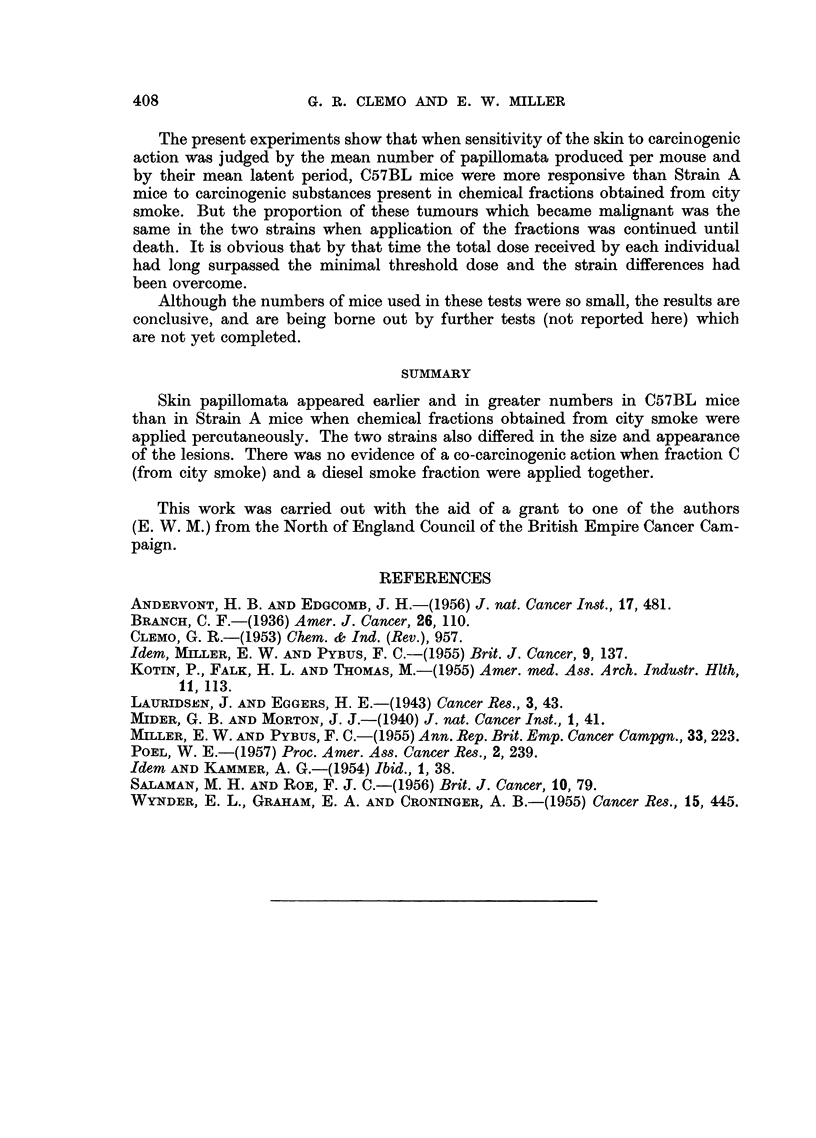# A Comparison of the Response of Two Inbred Strains of Mice to the Carcinogenic Action of City Smoke

**DOI:** 10.1038/bjc.1957.48

**Published:** 1957-09

**Authors:** G. R. Clemo, E. W. Miller


### A COMPARISON OF THE RESPONSE OF TWO INBRED STRAINS OF MICE TO THE CARCINOGENIC ACTION OF CITY SMOKE

G. R. CLEMO AND E. W. MILLER

*From Cherryburn, Mickley-on-Tyne, and the J. H. Burn Research Laboratory, Royal Victoria Infirmary, Newcastle upon Tyne*

Received for publication July 4, 1957

IN a previous communication (Clemo, Miller and Pybus, 1955) it was suggested that the epidermis of C57BL mice was more susceptible than that of Strain A mice to the carcinogenic action of fractions of city smoke applied percutaneously. A survey of the literature shows some disagreement on the relative merits of various inbred strains for skin tests for carcinogenicity. Thus, C57BL mice were more susceptible than Strain A (Branch, 1936) or C3H mice (Lauridsen and Eggers, 1943) to dibenzanthracene, but less susceptible than C57Br to methylcholanthrene (Mider and Morton, 1940), less susceptible than Swiss mice to cigarette tar (Wynder, Graham and Croninger, 1955) and less susceptible than Strain A to diesel exhaust products (Kotin, Falk and Thomas, 1955). Poel and Kammer (1954) found Strain C57BL to be the most susceptible of 3 strains to the percutaneous application of benzanthracene. Andervont and Edgcomb (1956) reported that their strain of C57BL mice was the most resistant of 7 inbred strains to the percutaneous application of methylcholanthrene and also was the only one to show deleterious effects ; they also found that the appearance of the skin lesion was characteristic for each strain. The relative responses probably depend on the carcinogen used and its strength, and on the particular sub-strain tested.

A further experiment has now confirmed that in this laboratory the C57BL mice are more susceptible than Strain A to skin carcinogens. It had already been shown that fraction C from city smoke (Clemo, 1953) was quite strongly carcinogenic (Clemo, Miller and Pybus, 1955) and that the second fraction obtained from diesel exhaust fumes was very weakly carcinogenic (Miller and Pybus, 1955) when painted on the skin of mice. So it was decided to combine the two fractions to discover whether there was any co-carcinogenic action. As this was one of a series of pilot experiments designed merely to test for the presence or absence of carcinogens, only a small number of mice was concerned.

### MATERIALS AND METHODS

Nine mice of Strain A (7 females and 2 males) and 10 of Strain C57BL (3 females and 7 males) were painted in the interscapular region with a benzene solution of fraction C plus the diesel fraction, a 1·0 per cent solution of each substance. The hair was not shaved and the solution was applied with 2 strokes of a No. 4 brush, 3 times weekly ; treatment was begun when the mice were 4 to 8 weeks old and was continued until death. The mice were killed when the tumours were judged to have become malignant. All apparently malignant and most apparently benign tumours were taken for histological examination.

The C57BL mice belonged to the eighth inbred generation (in this laboratory) of the C57BL/How strain, and the Strain A mice belonged to the A/$Gr_f$ ($F_7$) and $A_2G$ ($F_9$) strains ; no difference in the reactions of the two substrains of Strain A was detected. Comparison was made with the six C57BL/How ($F_5$) females painted with a 1·0 per cent solution of fraction C in the test already described (Clemo, Miller and Pybus, 1955).

A tumour was judged to be malignant when the cells were infiltrating through the panniculus carnosus ; in a number of cases where the tumour cells had not reached this muscle, although other signs of malignancy were present, the tumours were classed as " probably malignant ".

### RESULTS

Four C57BL males died of an intestinal infection after 6·5 to 7·0 months' treatment, before the earliest papilloma appeared, and are not included in the results. All but two of the Strain A mice died of pneumonia or kidney disease before their tumours had grown to any size, whereas all but two of the C57BL mice were killed on account of large malignant tumours. Only 3 strain A mice (No. 6, 7 and 9, Table I) had moderately large tumours and in only one of these (No. 7) did the tumour grow rapidly ; 4 of the C57BL mice had large tumours and only 2 had small skin tumours (No. 11, which died, and No. 14, which had to be killed on account of an osteogenic sarcoma of the spine which was obstructing the colon). Histological examination showed, however, that mere size was no criterion of malignancy.

TABLE I.—*The Response of Strain C57BL and Strain A Mice to the Percutaneous Appliation of Fraction C (from City Smoke) plus a Diesel Smoke Fraction*

| Strain | No. of mouse | Sex | Latent period of earliest papilloma (months) Individual | Mean | | Number of papillomata Maximum | Mean | Duration of treatment (months) Individual | Mean | | Mouse killed or died |
|---|---|---|---|---|---|---|---|---|---|---|---|
| A | 1 | F. | 10·5 | | | 2* | | 13·5 | | | K. |
| | 2 | „ | 12·5 | | | 1 | | 15·0 | | | D. |
| | 3 | „ | 13·5 | | | 3 | | 14·0 | | | „ |
| | 4 | „ | 13·5 | 12·5 | | 2 | | 14·0 | 14·0 | | „ |
| | 5 | „ | 15·0 | | | 1 | 1·9 | 15·5 | | | „ |
| | 6 | „ | 10·0 | | 11·6 | 2 | | 14·0 | | 13·3 | „ |
| | 7 | „ | 12·0 | | | 3 | | 12·0 | | | K. |
| | 8 | M. | 8·5 | 8·8 | | 2 | | 9·0 | 10·8 | | D. |
| | 9 | „ | 9·0 | | | 1 | | 12·5 | | | „ |
| C57BL | 10 | F. | 8·5 | | | 4 | | 11·0 | | | K. |
| | 11 | „ | 9·0 | 10·3 | | 3* | | 13·5 | 12·8 | | D. |
| | 12 | „ | 13·5 | | | 3 | | 14·0 | | | „ |
| | 13 | M. | 8·0 | | 9·3 | 2 | 3·2 | 10·5 | | 11·8 | K. |
| | 14 | „ | 8·5 | 8·2 | | 4 | | 9·5 | 10·7 | | „ |
| | 15 | „ | 8·0 | | | 3 | | 12·0 | | | „ |

* = one papilloma regressed.

The individual results are presented in Table I. The mean latent period for the development of the earliest papillomata was 11·6 months in Strain A and 9·3 months in Strain C57BL; this difference was significant ($d = 2·3$, $2\sigma_d = 2·19$). The average number of papillomata per mouse was 1·9 in Strain A and 3·2 in C57BL, again a significant difference ($d = 1{\cdot}3$, $2\sigma_d = 0{\cdot}28$). In each strain one papilloma regressed. The mean duration of treatment was 13·3 months in Strain A and 11·8 months in C57BL, and this time the difference was not significant ($d = 1{\cdot}5$, $2\sigma_d = 1{\cdot}95$). Most of the Strain A mice died from causes other than their tumours ; had they lived until the tumours were as large as those in the C57BL mice, the difference in duration of treatment might have become significant, but, as it turned out, some of the Strain A tumours, although small, were already malignant.

The final results, from the histological examination, showed (Table II) that there were 5 malignant tumours, one probably malignant, and 10 benign papillomata in Strain A ; one of the malignant tumours was a spindle-celled sarcoma (No. 1) and another (No. 9) a mixed sarcoma-epithelioma, the others being squamous epitheliomata. In Strain C57BL there were 4 malignant tumours (all squamous epitheliomata), 7 probably malignant, and 7 benign papillomata ; mouse No. 13 had a lung metastasis. When the final figures were compared it was found that the proportions of malignant tumours in the 2 strains were in agreement ; this was also the case when the " probably malignant " were included with the malignant tumours.

TABLE II.—*A Comparison of the Responses of Strain C57BL and Strain A Mice to the Percutaneous Application of Fraction C (City Smoke), Fractions C plus Diesel, and Fraction B (City Smoke)*

| Tested fraction | No. of mice strain and sex | Mean latent period of earliest papilloma (months) | Total No. of papillomata | Mean No. of papillomata | Mean duration of treatment (months) | Final result at death: Malignant | Probably Malignant | Benign |
|---|---|---|---|---|---|---|---|---|
| C | 6 C57BL F. | 8·7 | 16[1] | 2·7 | 12·0 | 7 | 3 | 4 |
| C plus | 6 C57BL F. & M. | 9·3 | 19 | 3·2 | 11·8 | 4 | 7 | 7 |
| Diesel | 9A F. & M. | 11·6 | 17 | 1·9 | 13·3 | 5 | 1 | 10 |
| B | 4 C57BL M. | 9·9 | 18[2] | 4·5 | 13·0 | 2 | 3 | 9 |
| | 6 A F. | 9·5 | 13[3] | 2·2 | 11·2 | 1 | 3 | 7 |

1 = 2 regressed.
2 = 7 coalesced to 3 in one mouse.
3 = 2 regressed.

These results show that papillomata appeared earlier and in greater numbers in C57BL mice than in Strain A mice, and that the resulting malignant tumours reached a greater size in C57BL mice, but that the same proportion of papillomata became malignant in the two strains. It cannot be stated from this experiment whether the tumours became malignant more rapidly in one strain or the other ; possibly, if some of the C57BL tumours had been sectioned earlier, they would have been found to be already malignant at a size comparable with those in Strain A.

In both strains papillomata appeared earlier in the males than in the females. In Strain A this difference was significant ($d = 3{\cdot}7$, $2\sigma_d = 1{\cdot}6$) but in Strain C57BL it was not ($d = 2{\cdot}1$, $2\sigma_d = 3{\cdot}2$). Duration of treatment was longer in females than in males in both strains (i.e. the females lived longer), but in neither strain was the difference significant (in Strain A $d = 3{\cdot}2$, $2\sigma_d = 3{\cdot}6$ ; in Strain C57BL, $d = 2{\cdot}1$, $2\sigma_d = 2{\cdot}4$).

A total of 17 lung adenomata appeared in the 9 Strain A mice (mean number per mouse = 1·9) and none in C57BL ; one lung nodule in the latter strain proved to be a metastasis from a skin epithelioma.

Owing to the definite strain difference, only the C57BL mice in this test could be compared with the 6 C57BL mice from the earlier experiment treated with fraction C only. The latter were 4 months old when first painted but, as shown in Table II, the average latent period for papillomata (8·7 months) was the same as in the mice treated with both fractions (9·3 months) ($d = 0{\cdot}6$, $2\sigma_d = 2{\cdot}1$) ; the mean number of papillomata per mouse (2·7 with fraction C, 3·2 with fraction C plus diesel) was the same in the two groups ; and the proportion of total tumours becoming malignant was also the same (50·0 per cent in those painted with fraction C only, 22·2 per cent in those painted with C plus diesel, $d = 27{\cdot}8$, 2 S.E. = 33·1), as was the proportion obtained when the "probably malignant" were added to the malignant (71·4 per cent with fraction C, 61·1 per cent with C plus diesel, $d = 10{\cdot}3$, 2 S.E. = 33·3). When fraction C and the diesel extract were combined there was, therefore, no enhancement of the carcinogenic effect due to fraction C alone.

In the previous communication (Clemo, Miller and Pybus, 1955) the results were presented in the form of a presence-or-absence of skin response to carcinogenic action, and the degree of response was not taken into account. Interesting comparative results are shown in Tables II and III where the numbers of papillomata are recorded for individual mice of the same two strains, A and C57BL, treated with fraction B in that experiment. The C57BL/Gr males were 2·5 months old and the Strain A females were 4·5 months old when treatment began. The mean latent period for the earliest papilloma was the same for the two strains, 9·9 and 9·5 months respectively. The mean number of skin papillomata per mouse was 4·5 in the C57BL mice and 2·2 in Strain A, a significant difference ($d = 2{\cdot}3$, $2\sigma_d = 2{\cdot}2$). The duration of treatment was significantly less in Strain A than in the C57BL mice ($d = 1{\cdot}8$, $2\sigma_d = 1{\cdot}6$) because, whereas the C57BL mice all developed large tumours before they died or were killed, the Strain A mice all died of the kidney disease characteristic of the strain while their tumours were

TABLE III.—*The Response of Strain C57BL and Strain A Mice to the Percutaneous Application of Fraction B (from City Smoke)*

| Strain | Sex | Latent period of earliest papilloma (months): Individual | Mean | Number of papillomata: Maximum | Mean | Duration of treatment (months): Individual | Mean |
|---|---|---|---|---|---|---|---|
| A | F. | 11·0 | 9·5 | 3 | 2·2 | 12·5 | 11·2 |
| | ,, | 8·5 | | 3[1] | | 12·0 | |
| | ,, | 10·5 | | 2 | | 10·5 | |
| | ,, | 8·0 | | 0 | | 8·0 | |
| | ,, | 11·0 | | 2 | | 12·5 | |
| | ,, | 8·0 | | 3[2] | | 11·5 | |
| C57BL | M. | 9·0 | 9·9 | 2 | 4·5 | 12·0 | 13·0 |
| | ,, | 11·0 | | 7[3] | | 13·0 | |
| | ,, | 8·5 | | 6 | | 13·5 | |
| | ,, | 11·0 | | 3 | | 13·5 | |

1 = one regressed.
2 = one regressed.
3 = coalesced to 3 tumours.

quite small. Nevertheless the proportions of tumours which became malignant were the same in the two strains. as were the proportions when the " probably malignant " tumours were added to the malignant. This bears out what has been stated above, that tumour size is no criterion of malignancy. The 6 Strain A mice developed 14 lung adenomata, but only one appeared in the 4 C57BL mice.

When the results obtained on C57BL mice with fraction B were compared with those obtained with fraction C (Table II), the mean latent periods were not significantly different, and the average numbers of papillomata per mouse were the same (4·5 for fraction B and 2·7 for fraction C, but $d = 1{\cdot}8$ and $2\sigma_d = 2{\cdot}7$). The duration of treatment was also the same, but more of the tumours produced by fraction C became malignant (50 per cent compared with 14·3 per cent ; $d = 35{\cdot}7$, 2 S.E.= 32·6) and the proportion was still significantly higher when the " probably malignant " were added to the malignant tumours (71·4 per cent, compared with 35·7 per cent ; $d = 35{\cdot}7$, 2 S.E.= 35·2). Therefore it is concluded that fraction C is more strongly carcinogenic than fraction B.

### DISCUSSION

Recently Poel (1957) has reported that more metastases occurred in female mice than in males of Strain C57 Leaden, when painted with 3,4-benzpyrene, and that, although the latent period for the development of papillomata was the same in the two sexes, malignant tumours developed more quickly from the papillomata in females and the final carcinomata were larger in the females. Using stock " S " mice painted with 9,10-dimethyl-1,2-benzanthracene and croton oil, Salaman and Roe (1956) obtained many more papillomata in males than in females, but the incidence of malignant tumours was the same in the two sexes. Andervont and Edgcomb (1956) found a sex-difference in the response of most of the 7 strains painted with methylcholanthrene but, whereas in Strain I the males were more susceptible, in Strain C57BL the females had a higher incidence of papillomata.

In the present test of fraction C plus diesel extract, where the numbers of mice were so small, there was no definite sex-difference in response within a strain except in one respect, namely that in Strain A the mean latent period for papillomata was less in the males than in the females. There was no significant difference between C57BL males and females in this respect, and otherwise, as regards the mean numbers of papillomata, the duration of treatment and the proportions of papillomata which became malignant, the two sexes of one strain showed the same response.

It has been shown by many workers in this field that sex-differences and strain-differences, which might not be apparent when higher doses are employed, can be demonstrated by the use of minimal doses of known carcinogens, or by sub-minimal doses and the use of croton oil or some other promoting agent. Working with unknown carcinogens of unknown concentration, it is just chance if such differences can be demonstrated.

Apart from the four C57BL mice which died early of an infection, the mice of this strain looked in far better health, especially in the later stages of the experiment, than did the Strain A mice. This is in contrast to the condition of the C57BL mice in the experiment of Andervont and Edgcomb (1956), where this was the only strain to show deleterious effects.

The present experiments show that when sensitivity of the skin to carcinogenic action was judged by the mean number of papillomata produced per mouse and by their mean latent period, C57BL mice were more responsive than Strain A mice to carcinogenic substances present in chemical fractions obtained from city smoke. But the proportion of these tumours which became malignant was the same in the two strains when application of the fractions was continued until death. It is obvious that by that time the total dose received by each individual had long surpassed the minimal threshold dose and the strain differences had been overcome.

Although the numbers of mice used in these tests were so small, the results are conclusive, and are being borne out by further tests (not reported here) which are not yet completed.

### SUMMARY

Skin papillomata appeared earlier and in greater numbers in C57BL mice than in Strain A mice when chemical fractions obtained from city smoke were applied percutaneously. The two strains also differed in the size and appearance of the lesions. There was no evidence of a co-carcinogenic action when fraction C (from city smoke) and a diesel smoke fraction were applied together.

This work was carried out with the aid of a grant to one of the authors (E. W. M.) from the North of England Council of the British Empire Cancer Campaign.

### REFERENCES

ANDERVONT, H. B. AND EDGCOMB, J. H.—(1956) *J. nat. Cancer Inst.*, **17**, 481.
BRANCH, C. F.—(1936) *Amer. J. Cancer*, **26**, 110.
CLEMO, G. R.—(1953) *Chem. & Ind.* (*Rev.*), 957.
*Idem*, MILLER, E. W. AND PYBUS, F. C.—(1955) *Brit. J. Cancer*, **9**, 137.
KOTIN, P., FALK, H. L. AND THOMAS, M.—(1955) *Amer. med. Ass. Arch. Industr. Hlth*, **11**, 113.
LAURIDSEN, J. AND EGGERS, H. E.—(1943) *Cancer Res.*, **3**, 43.
MIDER, G. B. AND MORTON, J. J.—(1940) *J. nat. Cancer Inst.*, **1**, 41.
MILLER, E. W. AND PYBUS, F. C.—(1955) *Ann. Rep. Brit. Emp. Cancer Campgn.*, **33**, 223.
POEL, W. E.—(1957) *Proc. Amer. Ass. Cancer Res.*, **2**, 239.
*Idem* AND KAMMER, A. G.—(1954) *Ibid.*, **1**, 38.
SALAMAN, M. H. AND ROE, F. J. C.—(1956) *Brit. J. Cancer*, **10**, 79.
WYNDER, E. L., GRAHAM, E. A. AND CRONINGER, A. B.—(1955) *Cancer Res.*, **15**, 445.